# Aesthetic and Functional Rehabilitation in Juvenile Ossifying Fibroma: A Case Report

**DOI:** 10.3390/reports8030122

**Published:** 2025-07-26

**Authors:** Nefeli Katanaki, Ioanna Pouliezou

**Affiliations:** 1 Division of Orthodontics, University of Geneva, 1205 Geneva, Switzerland; 2 Medical Research Methodology Unit, School of Medicine, Aristotle University of Thessaloniki, 54124 Thessaloniki, Greece; ipouliez@auth.gr

**Keywords:** juvenile ossifying fibroma, maxillary resection, prosthetic rehabilitation, pediatric oral tumor, long-term follow-up

## Abstract

**Background and Clinical Significance**: Juvenile ossifying fibroma (JOF) is a rare, benign, but locally aggressive fibro-osseous neoplasm that primarily affects the craniofacial skeleton of children and adolescents. Early surgical intervention is often required due to the lesion’s rapid growth and potential for significant facial deformity. Long-term functional and esthetic rehabilitation following maxillary resection in early childhood remains a clinical challenge. **Case Presentation**: This case reports a unique long-term follow-up of a 22-year-old female patient who underwent partial maxillary resection at the age of five due to JOF. Initial reconstructive efforts failed, necessitating a removable prosthesis to restore function and appearance. The patient experienced persistent self-consciousness and social withdrawal during adolescence, attributed to altered facial esthetics and repeated surgical disappointment. Nevertheless, prosthetic rehabilitation significantly improved mastication, phonetics, facial symmetry, and psychological well-being. **Conclusions**: The enduring psychosocial and functional impact of early maxillary resection for JOF and the pivotal role of prosthodontic management in long term rehabilitation are highlighted. A multidisciplinary approach that includes psychological support is suggested. This case report is among the few reports documenting long-term prosthetic outcomes for pediatric JOF patients extending into adulthood.

## 1. Introduction and Clinical Significance

Fibro-osseous lesions of the craniofacial bones are typically benign and slow-growing. Fibrous dysplasia, ossifying fibroma (OF), and cemento-ossifying dysplasia exhibit similar histopathological features to those observed in benign fibro-osseous lesions [1]. OF is a rare, benign, well-demarcated, and often encapsulated fibro-osseous tumor composed of fibrous tissue, metaplastic bone, and variable quantities of osteoid. It is subclassified into two clinicopathological subtypes: conventional and juvenile [2]. According to the World Health Organization (WHO) Classification of Head and Neck Tumors, OF are further divided into cemento-ossifying fibroma and juvenile ossifying fibroma (JOF), based on their clinical and histopathological characteristics [3]. While conventional OF generally affects individuals in their third or fourth decades of life, they exhibit slow progression and are usually managed by simple curettage, with low recurrence rates. Although they can occur at any age, the juvenile type is more aggressive, presents earlier in life, and has a significantly higher tendency for recurrence. JOF often manifests as a rapidly enlarging mass in patients aged 5 to 15 years, with well-defined radiographic margins and histological features consistent with OF, and is categorized as juvenile aggressive ossifying fibromas (JAOF). JOF typically manifests at an early age, with 79% of cases diagnosed before the age of 15. Originating from the periodontal ligament, JOF accounts for approximately 2% of oral tumors in the pediatric population [4].

JOF is a relatively rare fibro-osseous neoplasm [5] that primarily affects children and adolescents [1,2,4,6,7]. Most cases are diagnosed during the first or second decades of life. The maxilla is the most commonly involved site, although other craniofacial bones can also be affected [5,8]. Extragnathic involvement is rare but can affect the frontal or ethmoid bones [9]. Approximately 85% of cases occur mainly in the facial bones, 12% in the calvarium, and rarely, 3% extracranially. Nearly 90% of JOF are located in the facial region, with the majority affecting the paranasal sinuses and particularly the maxillary antra [10]. There is no gender predilection; males and females are equally affected [1,2,4,6,7,8,9]. Radiologically, the tumor appears as a well-circumscribed lesion, encased by a thin sclerotic bone shell. Despite being well-circumscribed, it is locally aggressive, disrupting cortical bone and invading multiple adjacent anatomical structures. Internally, the lesion is typically composed of soft tissue, demonstrating varying degrees of calcification or irregular linear bone formations. On computed tomography (CT) scans, JOF often appears as a low-density mass, due to cystic degeneration with diffuse enhancement observed following iodinated contrast intravenous administration.

Histologically, JOF is characterized by a highly cellular fibrous stroma, containing cementum particles and garland-shaped bony trabeculae [10]. Based on microscopic features, two histological variants are recognized: trabecular (TrJOF) and psammomatoid (PsJOF) [8]. Alternative terms include juvenile active ossifying fibroma [6,8,11], JOF [8,12,13], TrJOF, and trabecular desmo-osteoblastoma [8]. PsJOF more commonly develops in the cranial bones, whereas TrJOF predominantly affects the jaws. Accurate diagnosis requires a comprehensive approach that integrates clinical features, linear radiographic findings, CT imaging, and histopathologic evaluation [10].

OF accounts for approximately 3.1% of all oral tumors and 9.6% of all gingival lesions [14]. However, due to the limited number of reported cases in the literature, the incidence of JOF is difficult to estimate [15].

Clinically, TrJOF typically presents as a painless, progressive, and sometimes rapid expansion of the affected area. Maxillary involvement can provoke obstruction of the nasal passages and occasionally epistaxis [8]. On the contrary, PsJOF is clinically manifested as bone expansion often in the paranasal sinuses, particularly in the frontal and ethmoid areas [8,9,11,16]. Extension into the orbit can result in complications such as proptosis and visual complaints including blindness, nasal obstruction, ptosis, papilledema, and disturbances in ocular mobility [11,16,17].

## 2. Case Presentation

In April 2006, a 5-year-old female presented with a small erythematous exostosis located in the midline of the maxilla, leading her parents to seek dental consultation. By early May 2006, she was referred to a general hospital. The lesion showed rapid growth within days, with a change in color to a melanotic appearance. A preliminary diagnosis suggested a central maxillary lesion. By late May, a definitive diagnosis of JAOF was established and subsequently confirmed through histological examination. The lesion was found to be highly aggressive, involving the left maxilla, invading the nasal cavity, and expanding into the orbital cavity with associated bony resorption, imposing an extended surgical resection and, thus, an enlarged amputation. The findings from the pre-operative scintigraphy demonstrated the progression of the mass with two scans taken one month apart. Additionally, peripheral calcified granules were identified, allowing for qualitative radiographic differentiation of the lesion.

**Figure 1:**
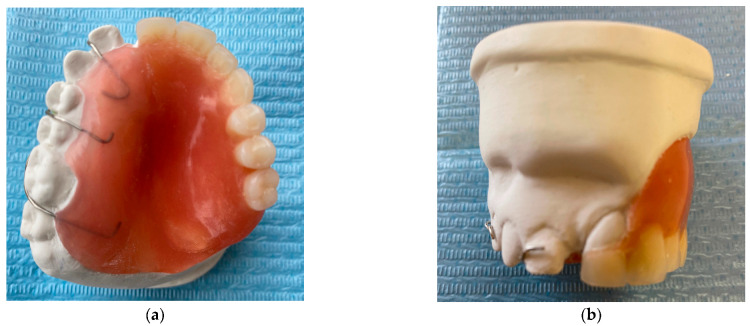
(**a**) Occlusal photograph of the previous prosthesis in dental cast. (**b**) Frontal photograph of the previous prosthesis in dental cast.

**Figure 2:**
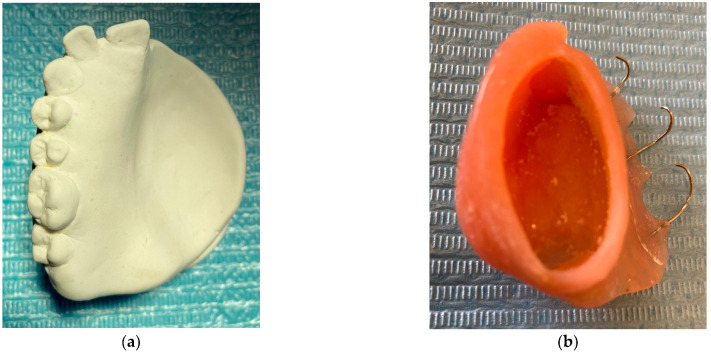
(**a**) Occlusal photograph of dental cast without the appliance. (**b**) The main characteristic of the removable appliance is the internal part that exceeds the maxillary sinus, which is hollow to achieve better retention with minimal weight.

**Figure 3:**
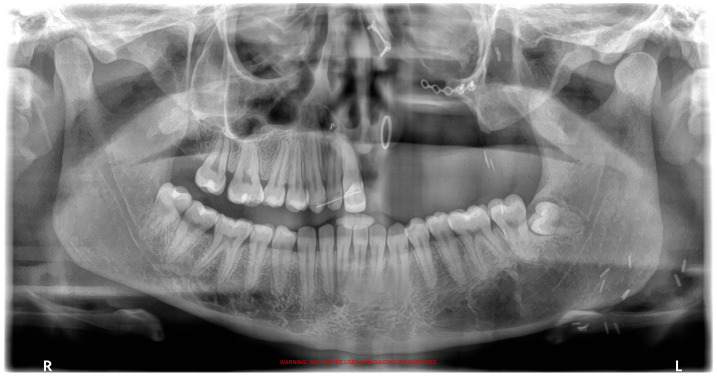
Panoramic radiograph after orthodontic treatment.

The fibroma was surgically excised. A bone graft harvested from the right scapula was placed in the defective area; however, due to the lack of vascularization, the graft was rejected. A second graft, taken from the left scapula, was also unsuccessful. Later on, a custom removable appliance was manufactured to restore function, esthetics, and facial contour of the stomatognathic system. The prosthesis on the dental cast is shown in Figure 1a, while Figure 1b shows the frontal view of the same cast. The occlusal anatomy of the edentulous defect is visualized in Figure 2a. The upper right central incisor was intruded, and a tooth extension of the removable appliance in #11 space was added. The appliance extended into the defective area, and an important feature was the hollow design of the segment extending into the sinus cavity, which contributed to increased retention while minimizing weight (Figure 2b). Over the course of time, the appliance had been periodically modified as needed to accommodate craniofacial growth and the development of neighboring structures. By October 2023, the patient had undergone orthodontic tooth movement with fixed appliances for space closure of the upper right quadrant. A panoramic radiograph was obtained, demonstrating the left-sided maxillary resection and completed orthodontic space closure, with preserved dental alignment on the contralateral side (Figure 3).

**Figure 4:**
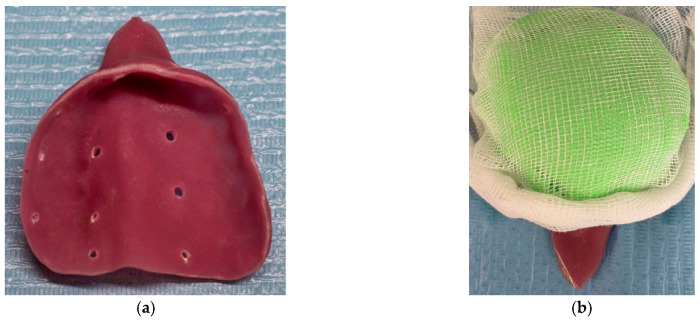
(**a**) Photograph of the acrylic custom tray. (**b**) Photograph of the acrylic custom tray loaded with alginate and gauze to prevent aspiration.

By late October of 2023, the patient attended a private dental practice due to significant loss of stability, support, retention, and esthetics of the appliance; thus, its replacement was deemed necessary. A custom impression tray was fabricated using the existing appliance as a template, with acrylic resin. Multiple perforations were drilled through the tray to enhance the retention of the impression material (Figure 4a). Alginate was then placed onto the tray, and a sterile gauze was placed over the surface to prevent aspiration of the material through the nasal cavity during impression taking. (Figure 4b). The impression was carried out successfully, and a working dental cast was fabricated. Both the cast and the previous appliance were forwarded to the dental laboratory for reference in designing the new prosthesis.

**Figure 5:**
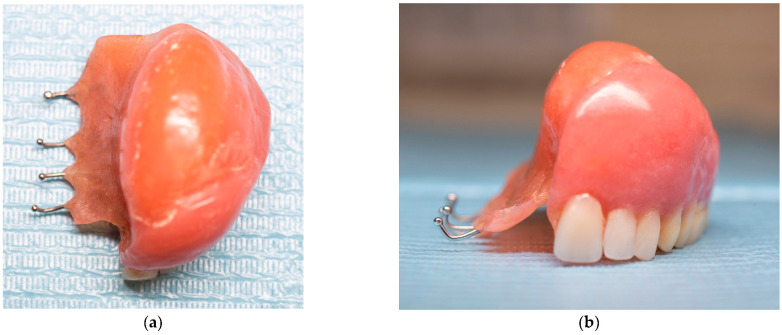
(**a**) Photographs of the new removable appliance that is replacing the left side of the patient’s maxillary space. The oval shape that is projected in the maxillary left sinus has a shallow groove that aids with the appliance’s retention. (**b**) Photograph of the appliance, which consists of the four metal clasps that support the prosthesis and are located in the space between the teeth.

**Figure 6:**
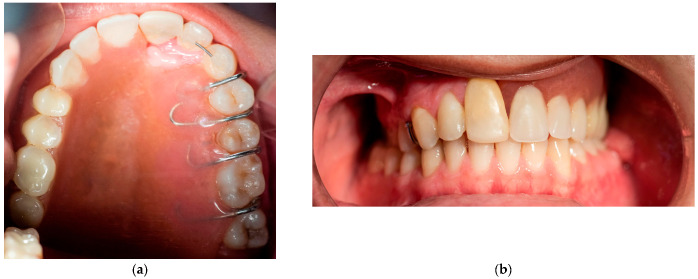
(**a**) Intraoral photograph of the upper occlusal arch. (**b**) Intraoral frontal photograph of appliance in situ.

The removable appliance was designed to restore the left side of the maxillary space defect. It replaced the base of the maxilla and extended into an oval projection that partially occupied the maxillary sinus space. This projection was bordered by a shallow peripheral groove, which played a critical role in the retention of the appliance (Figure 5a). The prosthesis was further stabilized by four 0.7 mm metal ball clasps, positioned in the interproximal spaces between the canine, premolars, and molars on the contralateral side of the defect (Figure 5b). Intraoral examination confirmed proper adaptation of the appliance, as demonstrated in the occlusal (Figure 6a) and frontal intraoral views (Figure 6b).

The design of the removable prosthesis was tailored to meet both anatomical and functional needs resulting from the maxillary resection. The hollow bulb configuration minimized the weight of the prosthesis while enhancing intra-sinus retention. The use of metal clasps provided mechanical stability on the unaffected side, compensating for the lack of support on the resected side. The prosthetic extension into the sinus was contoured to align with the internal morphology of the defect, with a peripheral groove to improve engagement and retention. Esthetic concerns were also addressed through carefully designing the anterior tooth extension. Overall, this design allowed the patient to regain mastication, speech articulation, and facial symmetry in the absence of surgical reconstruction.

The patient was informed with detailed instructions regarding the storage and hygiene of the removable prosthesis. A special designated container was provided for overnight storage. The patient was advised to clean the prosthesis daily with a soft toothbrush and water and to store it in water or a suitable cleansing solution during the night to maintain its integrity and hygiene.

**Figure 7:**
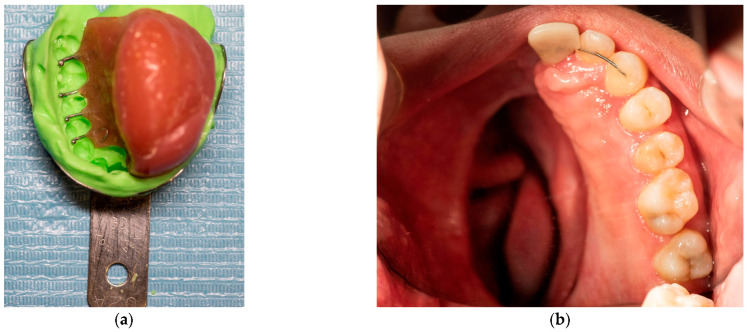
(**a**) Impression for clasp replacement. (**b**) Photograph of the intraoral upper occlusal arch.

A follow-up evaluation every 3 months was recommended to monitor the condition of the prosthesis and surrounding structures. Given the heavy masticatory forces exerted in the upper jaw quadrant, periodic replacement of one or more clasps was often required. Six months later, in the latest recall appointment in April 2024, as such cases normally require, an impression was made using a tray, while the appliance was placed in situ, and the impression was promptly sent to the laboratory for fabrication (Figure 7a). Figure 7b shows the edentulous arch without the prosthesis during a follow-up appointment, illustrating the healed soft tissue contours and defect margins.

**Figure 8:**
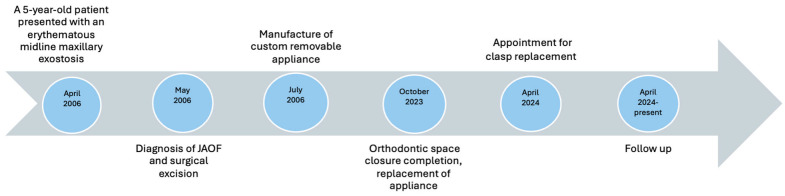
Case timeline of the diagnostic, surgical, orthodontic, and prosthetic milestones for this patient.

The importance of sufficient salivary flow was highlighted to the patient as it is necessary for the retention of the removable prosthesis in the oral cavity. She was informed that conditions or medications leading to hyposalivation should be closely monitored and appropriately managed. Although the patient had not undergone radiation therapy, they were educated about its potential impact on salivary gland function, should it become necessary in the future. This discussion aimed to reinforce the importance of salivary flow in retaining the removable prosthesis. A chronological overview of the diagnostic, surgical, orthodontic, and prosthetic milestones for this patient is provided in Figure 8.

## 3. Discussion

On gross examination, JOF are typically well-circumscribed and generally demonstrate a smooth surface. Similarly to conventional OF, intraoperative findings may reveal that the tumor “shells out” easily. However, certain lesions may appear more infiltrative in relation to the surrounding bone [8]. The TrJOF is characterized histologically by a cell-rich fibrous stroma containing bundles of cellular osteoid, which is often highly immature, and bone trabeculae without osteoblastic rimming with aggregates of giant cells [6,8]. On a microscopical level, TrJOF is unencapsulated and infiltrates the surrounding bone. Its stroma is loose and composed predominantly of spindle-shaped or polyhedral cells with minimal collagen production [1,2,7,8,9]. Histologically, the immature osteoid in some cellular immature osteoid form strands can be long and slender or plump. In some instances, distinguishing between the immature cellular osteoid and the cellular stroma can be challenging due to their similar cellular appearance [1,2,6,8,9]. Maturation into lamellar bone is typically absent, and irregular mineralization is commonly observed centrally within the osteoid strands. Additionally, localized aggregates of osteoclastic giant cells are often present within the stroma.

Another histologic feature that may be observed is mitotic activity within the stromal cells. Although this finding can raise concern for osteosarcoma, the number of mitotic figures is typically low and not indicative of malignancy [8]. On gross examination, both PsJOF and TrJOF variants of JOF usually appear yellowish-white and gritty. Under light microscopic examination, PsJOF is characterized by multiple small, round, and uniform ossicles, resembling psammomatoid bodies, embedded in a moderately cellular stroma composed of uniform, stellate, and spindle-shaped cells [8,16,18,19].

Radiographically, TrJOF initially presents as a unilocular radiolucent lesion. As the lesion enlarges, it often develops a multilocular appearance and causes progressive thinning of the cortical bone. CT imaging frequently reveals varying degrees of intralesional calcification, a feature strongly associated with the TrJOF. The degree of radiolucency or radiodensity depends on the quantity and distribution of the calcified tissue within the lesion [2,6,9,20]. In the case of PsJOF, the CT can show a characteristic “ground-glass’’ appearance, as well as multilocular or honeycomb patterns [9]. Additionally, PsJOF can present as a round, well-defined, sometimes corticated osteolytic lesion with a cystic morphology [8,16,17,18]. Notably, both the TrJOF and PsJOF tend to exhibit well-defined borders on imaging studies [5,7,8].

While these radiographic characteristics’ aid in clinical differentiation, the molecular profile of OF remains scarce. Only a limited number of molecular studies have investigated this group of lesions, and even fewer case reports are available in the literature. Some studies have identified mutations in HRPT2, a gene encoding the parafibromin protein [21]. These findings suggest a possible genetic contribution to tumor behavior, though further research is needed to establish molecular markers for prognosis or recurrence prediction. More recently, SATB2 gene rearrangements have been identified specifically in PsJOF, potentially distinguishing it from other subtypes [22]. Additionally, unlike in conventional OF, differential expressions of CDK4 and MDM2 have been observed in JOF, supporting molecular divergence between these lesions [23].

Management of JOF typically involves surgical intervention, including either curettage or surgical excision. The surgical approach depends on the size and anatomic site of the tumor. Small, well-contained tumors may be adequately treated with thorough curettage, whereas more extensive lesions often require en bloc resection or resection with reconstruction. Remarkably, JOF is associated with a high recurrence rate and an estimated range between 30% and 50% [5].

This case represents a rare, long-term follow-up of prosthetic management after early childhood maxillary resection for JOF, providing valuable insight into both the clinical workflow and the psychosocial adaptation of the patient treated. Beyond its clinical and surgical complexity, JOF can lead to profound psychosocial consequences, particularly when facial disfigurement occurs at a young age. The perception of facial esthetics is associated with personal identity and self-esteem, especially during adolescence, when social acceptance becomes paramount. In the present case, the early loss of maxillary structure and the associated facial asymmetry contributed to a persistent feeling of low self-confidence, which intensified during puberty. Despite initial surgical efforts and prosthetic rehabilitation, the patient’s self-consciousness, manifested by difficulty attending dental appointments, may be associated with emotional discomfort. Nevertheless, the removable prosthesis not only restored the oral function by establishing mastication and articulation but also significantly improved the patient’s facial profile, contributing to improved psychological well-being by restoring facial balance and enhancing their confidence in social interactions. In greater detail, the patient reported increased speech production and oral competence after receiving the prosthesis, highlighting the importance of managing functional impairments with esthetic rehabilitation.

Although surgical reconstruction is the treatment of choice, several case reports have demonstrated the efficacy of obturator prostheses in rehabilitating maxillary defects following JOF resection, particularly when reconstructive surgery is not feasible or fails. While the literature on pediatric cases is limited, existing reports support the use of various prosthetic designs, including hollow-bulb and clasp-retained obturators with favorable functional and esthetic outcomes [24,25,26,27,28,29,30,31,32,33,34,35,36,37]. Given the complexity of these cases, especially in young patients, effective management often requires a multidisciplinary approach, involving maxillofacial surgeons, prosthodontists, and psychological support teams, to address both anatomical and psychosocial rehabilitation.

Over the years, multiple adaptations were made to accommodate the patient’s growth and changing oral anatomy. During adolescence, several metal clasps were replaced or repositioned to improve retention as the dentition matured. Minor refinements were made to the contour of the bulb extension to reduce mucosal irritation. Additionally, the anterior tooth portion was reshaped on the two occasions to improve esthetics and phonetics in alignment with facial growth. After craniofacial maturity was reached, a definitive version of the prosthesis was fabricated with improved occlusal balance and long-term retention design. Follow-up care focused on maintaining clasp function, monitoring tissue response, and maintaining hygiene.

At 24 years of age, the patient had reached craniofacial maturity, allowing for stable, long-term prosthetic planning. The current management strategy focuses on regular clinical and radiographic monitoring to detect any potential recurrence of JOF. Periodic reassessment and maintenance of the removable prosthesis are essential to ensure continued functional and esthetic effectiveness, including modifications to improve retention, support, and hygiene as needed. Psychological follow-up is equally important to address the long-term emotional and social impact of early facial deformity. A multidisciplinary approach, including prosthodontics, maxillofacial surgery, and mental support, is essential to sustain the patient’s overall well-being and quality of life. Lifelong follow-up is recommended due to the potential for late recurrence. In this case, prosthetic rehabilitation not only restored oral function but also helped alleviate long-standing concern, contributing to improved self-confidence. Similar cases in the literature highlight the psychosocial value of obturator therapy in patients undergoing hemimaxillectomy [24,25].

## 4. Conclusions

JOF exhibits unique clinicopathological features. Accurate diagnosis requires careful correlation of clinical and radiographic findings with key histopathological features. Considering the different management approaches, establishing a correct tissue diagnosis is crucial to providing appropriate treatment. These tumors often demonstrate locally aggressive behavior, invading adjacent anatomical structures, which can complicate complete surgical removal and contribute to the high recurrence rate. Therefore, long-term clinical and radiographic follow-up is crucial, particularly for more aggressive lesions.

## Data Availability

The data supporting the findings of this case report are available from the corresponding author upon reasonable request.

## Abbreviations

The following abbreviations are used in this manuscript:

CT	Computed tomography
JAOF	Juvenile aggressive ossifying fibroma
JOF	Juvenile ossifying fibroma
OF	Ossifying fibroma
PsJOF	Psammomatoid juvenile ossifying fibroma
TrJOF	Trabecular juvenile ossifying fibroma
WHO	World Health Organization
